# Single center clinical analysis of macrophage activation syndrome complicating juvenile rheumatic diseases

**DOI:** 10.1186/s12969-024-00991-3

**Published:** 2024-05-23

**Authors:** Shuoyin Huang, Yingying Liu, Wu Yan, Tonghao Zhang, Panpan Wang, Meifang Zhu, Xiaohua Zhang, Peng Zhou, Zhidan Fan, Haiguo Yu

**Affiliations:** 1 Department of Rheumatology and Immunology, Children’s Hospital of Nanjing Medical University, Nanjing, 210008 China; 2 Department of Child Health Care, Children’s Hospital of Nanjing Medical University, Nanjing, China

**Keywords:** Macrophage activation syndrome, Systemic juvenile idiopathic arthritis, Kawasaki disease, Systemic lupus erythematosus, Rheumatic diseases, Interleukin (IL)-18, Ferritin

## Abstract

**Background:**

Macrophage activation syndrome (MAS), an example of secondary hemophagocytic lymphohistiocytosis, is a potentially fatal complication of rheumatic diseases. We aimed to study the clinical and laboratory characteristics, treatment schemes, and outcomes of different rheumatic disorders associated with MAS in children. Early warning indicators of MAS have also been investigated to enable clinicians to make a prompt and accurate diagnosis.

**Methods:**

Fifty-five patients with rheumatic diseases complicated by MAS were enrolled between January 2017 and December 2022. Clinical and laboratory data were collected before disease onset, at diagnosis, and after treatment with MAS, and data were compared between patients with systemic juvenile idiopathic arthritis (sJIA), Kawasaki disease (KD), and systemic lupus erythematosus (SLE). A random forest model was established to show the importance score of each variable with a significant difference.

**Results:**

Most (81.8%) instances of MAS occurred during the initial diagnosis of the underlying disease. Compared to the active stage of sJIA, the platelet count, erythrocyte sedimentation rate, and fibrinogen level in sJIA-MAS were significantly decreased, whereas ferritin, ferritin/erythrocyte sedimentation rate, aspartate aminotransferase, alanine aminotransferase, lactate dehydrogenase, and D-dimer levels were significantly increased. Ferritin level, ferritin/erythrocyte sedimentation rate, and platelet count had the greatest predictive value for sJIA-MAS. The level of IL-18 in the sJIA-MAS group was significantly higher than in the active sJIA group, whereas IL-6 levels were significantly lower. Most patients with MAS were treated with methylprednisolone pulse combined with cyclosporine, and no deaths occurred.

**Conclusions:**

Thrombocytopenia, ferritin levels, the ferritin/erythrocyte sedimentation rate, and elevated aspartate aminotransferase levels can predict the occurrence of MAS in patients with sJIA. Additionally, our analysis indicates that IL-18 plays an important role in the pathogenesis of MAS in sJIA-MAS.

## Background

Macrophage activation syndrome (MAS) is a subtype of hemophagocytic lymphohistiocytosis (HLH), which can cause multisystem failure and death as a complication of rheumatic immune diseases [1]. Systemic JIA is complicated by MAS -incidence of 10%, and MAS occurs at a subclinical level in 30–40% patients [2]. SLE, KD can be complicated by MAS, with incidence of 0.9–4.6% and 1.1%, respectively. MAS can also be observed in other subtypes of juvenile idiopathic arthritis, dermatomyositis, periodic fever, vasculitis and autoinflammatory diseases [3]. Previous studies have reported that the mortality rate of sJIA-MAS ranges from 8 to 23% [4], while that of SLE-MAS ranges from 5 to 35% [5–7].

The etiology and pathogenesis of MAS remain unclear. Chronic inflammation (such as during sJIA or SLE), genetic susceptibility and/or infection, drugs, and other factors trigger the activation of mononuclear macrophages, which produce large quantities of cytokines such as IL-1, IL-18, IFN-γ, and others, creating a cascade reaction between immune cells and cytokines, leading to a cytokine storm which represents the main cause of this disease [8]. Previous studies have found that heterozygous mutations in some genes may be associated with the pathogenesis of MAS [9–11]. The characteristics of MAS include fever; enlargement of liver, spleen, and lymph nodes; central nervous system (CNS) dysfunction; bleeding manifestations; pancytopenia; liver function impairment; hyperferritinemia; and coagulation impairment [12].

Many overlapping clinical and laboratory features between MAS and primary diseases, make it more challenging to differentiate MAS. Patients with active sJIA are persented with fever, elevated ferritin levels, hepatosplenomegaly, and lymphadenopathy. In addition, patients with active SLE are obversed with fever, cytopenias and elevated ferritin levels [13]. The aim of this study was to describe the clinical and laboratory features, treatments, and outcomes of MAS, and to compare the differences in the features of sJIA, KD, and SLE in conjunction with MAS. A random forest model was used to analyze the risk factors associated with sJIA-MAS.

## Methods

### Patients

We retrospectively analyzed the electronic medical records of patients diagnosed with MAS at Nanjing Children’s Hospital between January 2017 and December 2022. A total of 55 patients with an initial diagnosis of MAS were recruited. The underlying diseases included sJIA-MAS (*n* = 34), KD-MAS (*n* = 10), and SLE-MAS (*n* = 11). The diagnosis of sJIA was based on the classification criteria of the International Federation of Rheumatology Societies [14]. Diagnosis of KD was based on the guidelines of the American Heart Association [15]. SLE criteria was in 2012, the Systemic Lupus International Collaborating Clinics (SLICC) proposed revised classification criteria [16]. MAS primarily refers to the initial diagnostic guidelines proposed by Ravelli et al. in 2016 [17]. In addition, the diagnosis of SLE-MAS refers to the criteria proposed by Parodi et al. in 2009 [18]. Other related diseases such as infectious diseases, other immune diseases, tumors, hematological diseases, and inherited metabolic diseases were excluded.

### Data collection

The following information was collected from the included cases: (1) demographic data including sex, age of onset for the underlying diseases and MAS; (2) clinical characteristics of MAS including fever, hepatomegaly, splenomegaly, hemorrhage, neuropsychiatric symptoms, etc.; (3) laboratory data collected before the onset of MAS (pre-MAS), during the onset of MAS, and after MAS treatment, include those known to be most relevant for MAS diagnosis, such as ferritin, platelets, erythrocyte sedimentation rate, aspartate aminotransferase, alanine aminotransferase, etc.; (4) treatment and prognosis of MAS; (5) The hallmark of MAS is an uncontrolled immune response involving continual activation and expansion of T lymphocytes and macrophages, resulting in marked hypercytokinaemia. Therefore, our research team used ELISA to detect the expression levels of IL-6, IL-18, SLR-2R, IL-10 and TNF-a before and after MAS.

### Statistical analysis

Quantitative data are presented as medians and interquartile ranges, and categorical data are presented as frequencies and percentages. Categorical data were compared using the chi-squared or Fisher’s exact tests. The Friedman test or Kruskal–Wallis test was used to compare descriptive data between different groups, as appropriate. Pairwise comparisons and Bonferroni corrections were used to compare different groups. The random forest model was used to compare the importance scores of the related risk factors of the sJIA-MAS (single factor, *p* < 0.05), and the area under the ROC curve was used to express the predictive value. Statistical significance was determined if *p* < 0.05. Statistical analyses were performed using SPSS software (version 27.0; IBM Corp., Armonk, NY, USA).

## Results

A total of 55 patients with MAS were admitted to the Department of Rheumatology and Immunology at our hospital between January 2017 and December 2022, including 34 patients with sJIA-MAS, 10 with KD-MAS, and 11 with SLE-MAS. This study included 36 females and 19 males, demonstrating that there were more women than men with MAS and different rheumatic diseases. The median age at onset for these three diseases was 5.8, 1.3, and 11.1 years, respectively. Most MAS cases occurred at the initial diagnosis of the underlying diseases, with proportions of 82.4%, 100%, and 63.6%, respectively (Table 1).

**Table 1:** Demographic characteristics of all patients at the onset of MAS. (*n* = 55)

Characteristics	sJIA (*n* = 34)	KD (*n* = 10)	SLE(*n* = 11)
Female, n (%)	21(62.8)	7(70.0)	8(80.0)
Age at onset of underlying disease(y), median (IQR)	5.8(2.6–10.9) †	1.3(0.4–4.8) *	11.1(9.8–12.3) ‡
Age at onset of MAS(y), median (IQR)	6.9(3.4–10.9) †	1.3(0.4–4.8) *	11.1(9.8–14.8) ‡
MAS onset during first diagnosed underlying disease, n(%)	28(82.4)	10(100)	7(63.6)

sJIA systemic juvenile idiopathic arthritis, KD Kawasaki disease, SLE systemic lupus erythematosus, MAS macrophage activation syndrome, IQR interquartile range.*P < 0.05 compared to sJIA group, †*P* < 0.05 compared to SLE group, ‡*P* < 0.001 compared to KD group

Fever at MAS onset was observed in 96.3% (53/55) of patients. Two children with sJIA-MAS had jaundice and severe hepatitis as initial symptoms, and fever developed during disease progression. A total of 90.9% (50/55) of children had rashes. sJIA-MAS was more likely to involve the interstitial lung tissue (4/34, 11.8%). Compared to patients with sJIA-MAS and KD-MAS, patients with SLE-MAS had a higher proportion of CNS involvement (81.8%). The incidence of serosal effusion was also significantly higher in the SLE-MAS group (63.6%). Hypotension occurred in eight patients at the time of MAS. The incidence of hypotension in patients with KD-MAS was as high as 30%. Most (52 of 55) patients with MAS underwent bone marrow aspiration. The positive rates of phagocytes in sJIA-MAS, KD-MAS, and SLE-MAS were 42.4%, 75%, and 81.8%, respectively (Table 2).

**Table 2:** Clinical features of all patients at the onset of MAS. (*n* = 55)

	Feature	sJIA (*n* = 34)	KD (*n* = 10)	SLE(*n* = 11)
1	Fever	33(97.1)	10(100)	10(90.9)
2	Rash	33(97.1)	6(60.0) *	11(100) **‡**
3	Hepatomegaly	28(61.8)	7(70.0)	7(63.6)
4	Splenomegaly	16(38.2)	1(10.0)	4(36.4)
5	Lymphadenopathy	29(79.4)	7(70.0)	7(63.6)
6	Arthralgia/arthritis	20(58.8)	7(70.0)	3(27.3)
7	CNS involvement	9(26.5) **†**	6(60.0)	9(81.8)
8	Lethargy	3(8.8) **†**	5(50.0) *	5(45.4)
9	Seizures	1(2.9) **†**	1(10.0)	3(27.3)
10	Irritability	1(2.9)	2(20.0)	1(9.1)
11	Confusion	0(0)	1(10.0)	0(0)
12	Headache	1(2.9)	0(0)	1(9.1)
13	Mood changes	1(2.9)	0(0)	0(0)
14	Coma	0(0)	0(0)	0(0)
15	Heart involvement	4(11.8)	5(50.0) *	3(27.3)
16	Hypotension	2(5.9)	3(30.0)	3(27.3)
17	Coronary enlargement	0(0)	1(10)	0(0)
18	Arrhythmia	1(2.9)	0(0)	0(0)
19	Heart failure	0(0)	0(0)	0(0)
20	Cardiomegaly	0(0)	0(0)	0(0)
21	Lung involvement	19(55.8) **†**	5(50.0)	1(9.1)
22	Respiratory failure	0(0)	0(0)	0(0)
23	Pneumonia	15(33.3) **†**	5(50.0)	0(0) **‡**
24	Interstitial infltrates	4(11.8)	0(0)	1(9.1)
25	Pleurisy	0(0)	0(0)	0(0)
26	Pulmonary hemorrhage	0(0)	0(0)	0(0)
27	Acute respiratory distress syndrome	0(0)	0(0)	0(0)
28	Hemorrhagic manifestations	7(20.6)	1(10.0)	1(9.1)
29	Petechiae, ecchymosis or purpura	2(5.9)	0(0)	0(0)
30	Mucosal bleeding	3(8.8)	0(0)	0(0)
31	Gastrointestinal bleeding	1(2.9)	1(10.0)	0(0)
32	Epistaxis	3(8.8)	0(0)	1(9.1)
33	Intravascular coagulation	0(0)	0(0)	0(0)
34	Intracranial hemorrhages	0(0)	0(0)	0(0)
35	Gastrointestinal involvement	2(5.9)	2(20.0)	0(0)
36	Abdominal distention/pain	2(5.9)	2(20.0)	0(0)
37	Gastroenteritis	0(0)	1(10.0)	0(0)
38	Kidney involvement	2(5.9) **†**	0(0)	10(90.9) **‡**
39	Renal failure	2(5.9)	0(0)	0(0)
40	Proteinuria	0(0) **†**	0(0)	10(90.9) **‡**
41	Hematuria	0(0) **†**	0(0)	10(90.9) **‡**
42	Serous cavity efusion	10(29.4)	2(20.0)	7(63.6)
43	Bone marrow aspirate	14(42.4)	6(75.0)	9(81.8)

*sJIA* systemic juvenile idiopathic arthritis, *KD* Kawasaki disease, *SLE* systemic lupus erythematosus, *MAS* macrophage activation syndrome, *CNS* central nervous system. *P < 0.05 compared to sJIA group, †*P* < 0.05 compared to SLE group, ‡*P* < 0.05 compared to KD group

Compared to the active sJIA group, the platelet count, erythrocyte sedimentation rate (ESR), and fibrinogen levels in the sJIA-MAS group were significantly decreased (*P* < 0.05), whereas the ferritin, ferritin/ESR, aspartate aminotransferase (AST), alanine aminotransferase (ALT), sodium lactate dehydrogenase, and D-dimer levels were significantly increased (*P* < 0.05) (Table 3). Almost all patients with KD-MAS and SLE-MAS had abnormal liver function and elevated ferritin levels. The ferritin levels and ferritin/ESR ratios of patients with sJIA-MAS were significantly higher than those of patients with KD-MAS and SLE-MAS (*P* < 0.05) (Table 4).

**Table 3:** Laboratory values of patients with sJIA at pre-MAS, onset of MAS and after treatment of-MAS. (*n* = 34)

Laboratory variables	pre-MAS		post-MAS		Post treatment-MAS	
	*n*	Median(IQR)	*n*	Median(IQR)	*n*	Median(IQR)
WBC (×109/L)	33	14.47(10.25–20.78)	34	7.76(5.54–16.32)	34	12.6(8.83–23.03)
Hb (g/L)	33	104(98.5-112.5)	34	101(92–112)	34	104.5(96-111.8)
PLT (×109/L)	33	258(210–396)*	34	136(99.75–237.8)	34	391.5(215–472)
CRP (mg/L)	32	64.5(25-97.25)	34	25.5(8-62.5)	34	8(8–12)
ESR (mm/h)	29	64(40-85.5)*	28	30(18.25–37.83)	28	16.5(8.25–35.5)
Alb (g/L)	28	35.05(32.8537.83)	34	33.5(31.78–36.18)	34	37.7(35.1-41.55)
ALT (U/L)	32	28.85(13-135.5)*	34	281(61.75–698.3)	34	110.5(43.75–205.3)
AST (U/L)	32	45(26.5-91.25)*	34	191(83.75–541.5)	34	33(24.75–56.25)
LDH (U/L)	29	472(302–611)*	34	825.5(609–1420)	34	382.5(301.8-492.3)
TG (mmol/L)	28	1.40(1.17–2.03)	34	2.04(1.48–2.42)	34	2.36(1.56–3.55)
Dimer (mg/L)	22	1174(584–2517)*	28	2199(664.3–7280)	27	292(180–521)
FIB (g/L)	25	3.58(2.82–4.89)*	31	2.43(1.6–2.71)	30	2.13(1.43–3.87)
SF (µg/L)	29	1500(854.5–2146)*	34	11,014(5546–22,930)	34	1887(740.5–3637)
SF/ESR	28	24.98(15.76–40.95)*	27	411.1(106.6-709.3)	28	89.41(32.75–130.3)

*sJIA* systemic juvenile idiopathic arthritis, *MAS* macrophage activation syndrome, *WBC* white blood cell, *Hb* Hemoglobin, *PLT* platelet count, *CRP* C-reactive protein, *ESR* erythrocyte sedimentation rate, *Alb* albumin, *ALT* alanine aminotransferase, *AST* aspartate aminotransferase, *LDH* lactate dehydrogenase, *TG* triglycerides, *FIB* fibrinogen, *SF* serum ferritin, *IQR* interquartile range, *n* number of patients with laboratory data available. * *P* < 0.05 compared with post-MAS

**Table 4:** Laboratory values of all patients at MAS. (*n* = 55)

Laboratory test	sJIA(*n* = 34)		KD(*n* = 10)		SLE(*n* = 11)		
	*n*	Median(IQR)	*n*	Median(IQR)		*n*	Median(IQR)
WBC (×109/L)	34	7.76(5.54–16.32)*	8	25.2(7.8–30.9)†		11	3.26(1.7–6.29)‡
Hb (g/L)	34	101(92–112)	8	101.0(79.3–118.0)		11	106(86.0-112.0)
PLT (×109/L)	34	136(99.75–237.8)	8	104.5(81.8-226.8)		11	126(101.0-184.0)
CRP (mg/L)	34	25.5(8-62.5)	8	46.5(15.8–128.0)†		11	8(8.0–25.0)‡
ESR (mm/h)	28	30(18.25–37.83)	8	27.5(14.3–92.0)		9	34(13.0-54.5)
Alb (g/L)	34	33.5(31.78–36.18)*	8	27.5(25.4–32.9)†		11	32.6(32.3–39.2)
ALT (U/L)	34	281(61.75–698.3)*	8	75.5(45.5–156.0)†		11	114(35.0-261.0)
AST (U/L)	34	191.7(83.7-541.5)*	8	32.0(25.5–82.5)†		11	62(28.0-219.0)‡
LDH (U/L)	34	825.5(609–1420)*	8	467.0(423.5–552)†		11	1137(450.0-1324.0)
TG (mmol/L)	34	2.04(1.48–2.42)	8	2.0(1.4–2.3)		11	2.4(1.65–4.36)
D-dimer (mg/L)	28	2199(664.3–7280)	7	1471.0(823–5680)		9	4430(619.0-6857.0)
FIB (g/L)	31	2.43(1.6–2.71)	7	2.1(1.4–3.3)		10	2.37(1.818–3.195)
SF (µg/L)	34	11,014(5546–22,930)*	7	1178(360.2–3212)†		11	2724(1468–4600.0)‡
SF/ESR	27	411.1(106-709.3)*	7	17.3(7.6-247.1)†		9	121.1(58.74–421.8)‡

*sJIA* systemic juvenile idiopathic arthritis, *KD* Kawasaki disease, *SLE* systemic lupus erythematosus, *MAS* macrophage activation syndrome, *WBC* white blood cell, *Hb* Hemoglobin, *PLT* platelet count, *CRP* C-reactive protein, *ESR* erythrocyte sedimentation rate, *Alb* albumin, *ALT* alanine aminotransferase, *AST* aspartate aminotransferase, *LDH* lactate dehydrogenase, *TG* triglycerides, *FIB* fibrinogen, *SF* serum ferritin, *IQR* interquartile range, *n* number of patients with laboratory data available* *P* < 0.05 compared with KD group, † *P* < 0.05 compared with SLE group, ‡ *P* < 0.05 compared with sJIA group

**Fig. 1:**
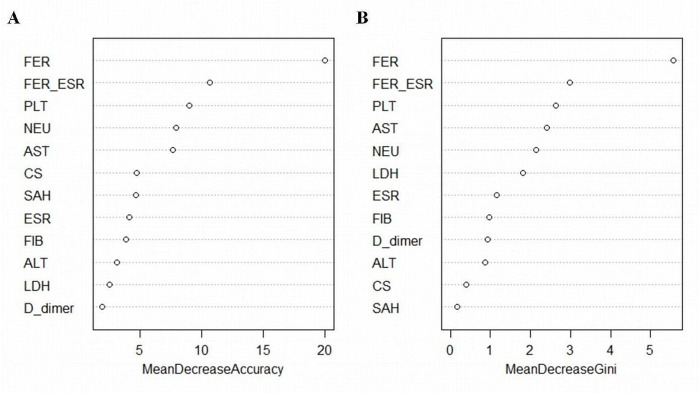
Graph of random forest algorithm. The horizontal axis is the importance score of the variable, and the vertical axis is each variable in the model. The higher the score, the greater the influence of the corresponding variable on the classification of the model. FER ferritin, ESR erythrocyte sedimentation rate, PLT platelet count, AST aspartate aminotransferase, Neu neutrophil, LDH lactate dehydrogenase, FIB fibrinogen, ALT alanine aminotransferase, CS Central system involvement, SAH Systemic hemorrhage

**Fig. 2:**
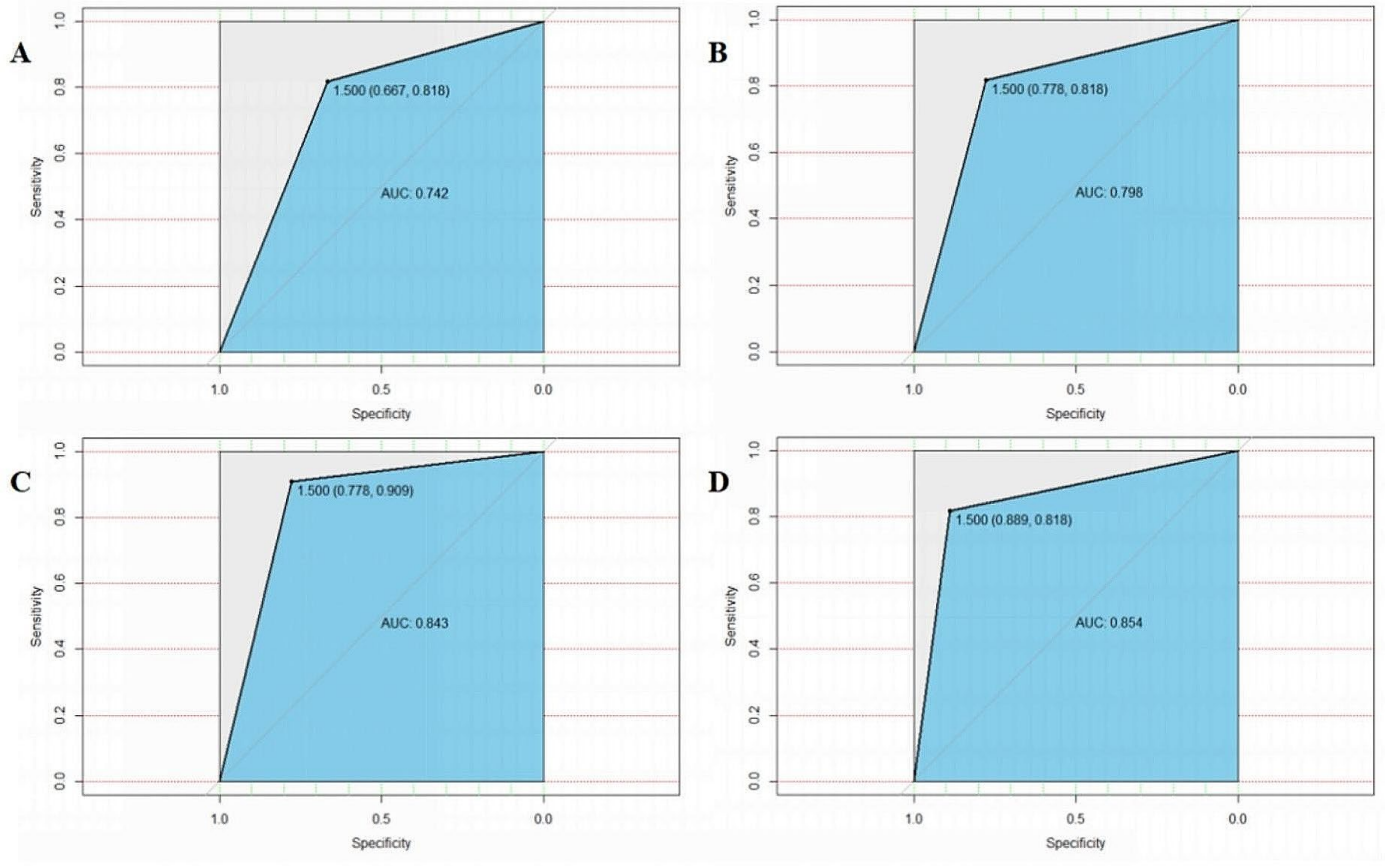
ROC curve of random forest algorithm. (A) the AUC of SF, SF/ESR and PLT in the combination of MAS diagnosis was A0.742; (B) The AUC of the combination of SF, SF/ESR, PLT, AST, NEU and AST in the diagnosis of MAS was 0.789; (C) The AUC of the combination of SF, SF/ESR, PLT, AST, NEU, LDH, ESR, FIB and D-dimer in the diagnosis of MAS was 0.843. (D) The AUC of the combination of SF, SF/ESR, PLT, AST, NEU, LDH, ESR, FIB, D-dimer, ALT, CS and SAH in the diagnosis of MAS was 0.854

The random forest model was used to analyze the risk factors related to sJIA-MAS. The sJIA-MAS-related risk factors (single factor *p* < 0.05) were included, and the importance of each variable in the model was scored (Fig. 1). The importance of the predictors in the model was ranked as follows: ferritin, ferritin/ESR, platelet counts, AST, neutrophils, lactate dehydrogenase, ESR, fibrinogen, D-dimer, ALT, central nervous system manifestation, and bleeding manifestations. The area under the ROC curve is the result of a comprehensive evaluation. In this study, ferritin level, ferritin/ESR, and platelet count had the greatest predictive value for MAS. The area under the ROC curve for the combination of these three factors was as high as 0.742, illustrating that the prediction performance was good. The areas under the ROC curve of the top six, top nine, and top twelve combined were 0.798, 0.843, and 0.854, respectively (Fig. 2).

**Fig. 3:**
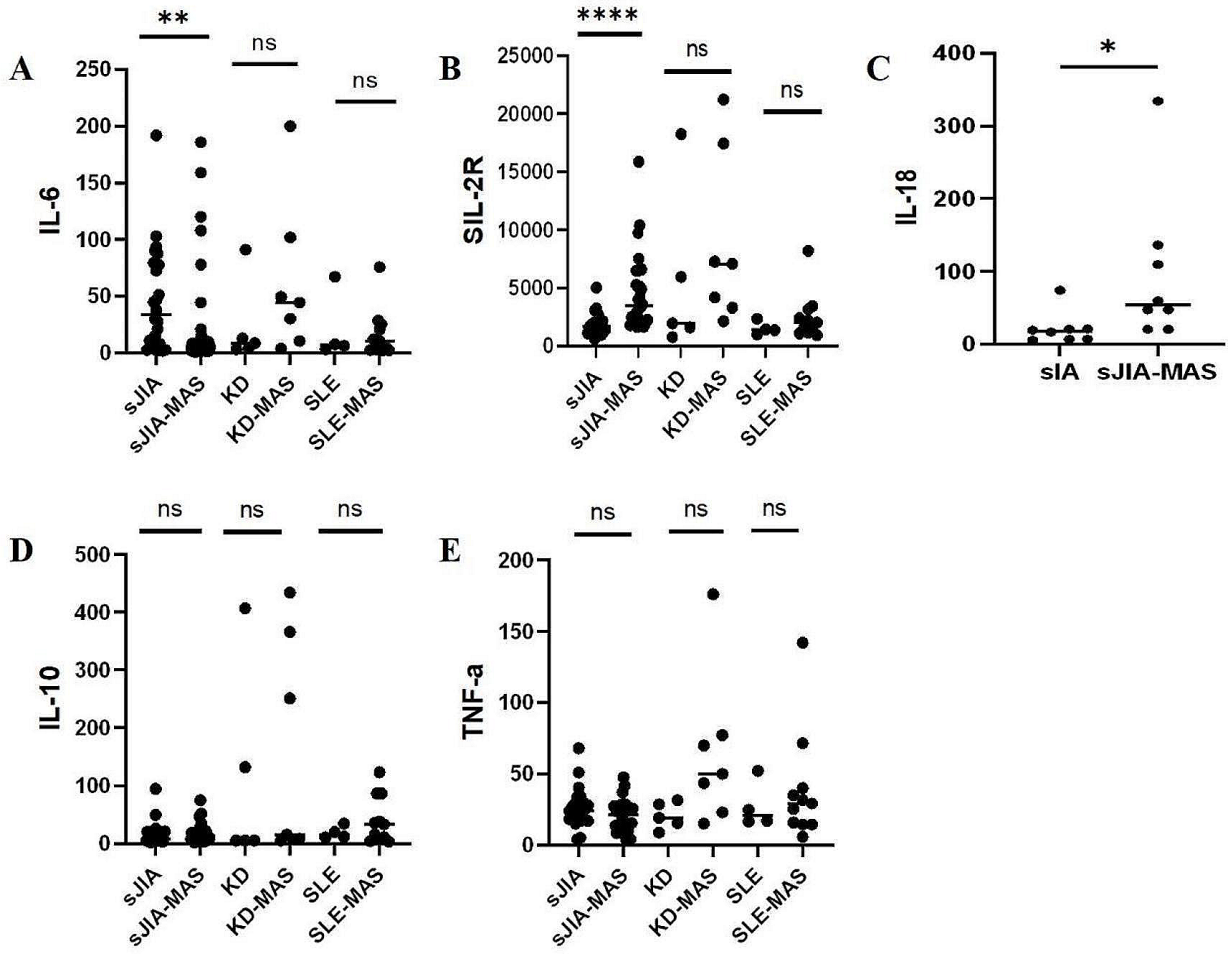
cytokine profile of (**A**) IL-6, (**B**) sIL-2R, (**C**) IL-18, (**D**) IL-10 and (**E**) TNF-α. *P<0.05, **P<0.01, ***P<0.001

The expression levels of IL-6, IL-18, SLR-2R, IL-10 and TNF-a were detected by ELISA. The IL-6 levels in patients with sJIA-MAS were significantly lower than those in patients with active sJIA (*P* < 0.01). IL-18 levels in patients with sJIA-MAS were significantly higher than those in patients with active sJIA (*P* < 0.05). The sJIA-MAS sIL-2R level was significantly higher than that of sJIA during the active stage (*P* < 0.001) (Fig. 3).

All patients received glucocorticoid therapy and 89.0% (49/55) received methylprednisolone pulse therapy. Overall, 61.8% (34/55) of patients were treated with cyclosporine: 34 in the sJIA-MAS group, 2 in the KD-MAS group, and 7 in the SLE-MAS group. Four patients with sJIA-MAS and three with SLE-MAS were treated with intravenous immunoglobulin (IVIG). Plasma exchange was performed in one patient with SLE-MAS, while another patient with SLE-MAS was treated with cyclophosphamide (Table 5).

**Table 5:** Therapeutic interventions and outcomes of all patients with MAS. (*n* = 55)

Therapeutic intervention	SJIA (*n* = 34)	KD (*n* = 10)	SLE(*n* = 11)	Total (*n* = 55)
Any corticosteroids	34(100)	10(100)	11(100)	55(100)
Pulse methylprednisolone	29(85.3)	6(60)	10(90.9)	49(89.0)
Cyclosporine	25(73.5)	2(20)*	7(63.3)	34(61.8)
Intravenous immunoglobulin	4(11.7)	10(100)*	3(27.2)‡	17(30.9)
Biologic medications	4(11.7)	0(0)	1(9.0)	5(9.0)
Tocilizumab	4(11.7)	0(0)	0(0)	4(7.20)
Rituximab	0(0)	0(0)	1(9.0)	1(1.8)
Cyclophosphamide	0(0)	0(0)	1(9.0)	1(1.8)
Plasmapheresis	0(0)	0(0)	1(9.0)	1(1.8)

*sJIA* systemic juvenile idiopathic arthritis, *KD* Kawasaki disease, *SLE* systemic lupus erythematosus, *MAS* macrophage activation syndrome, *P < 0.05 compared to sJIA group. ‡*P* < 0.05 compared to KD group

## Discussion

MAS is a life-threatening complication of rheumatic disease. Although many classification and diagnostic criteria have been published to distinguish MAS, the early diagnosis of MAS is still a challenge. In our study, almost all patients (53/55, 96.3%) had persistent high fever at the time of MAS diagnosis. When the fever type in sJIA changes from intermittent fever to persistently high fever that is difficult to relieve, the occurrence of MAS should be monitored [6]. The children with sJIA-MAS, close attention to subtle pulmonary symptoms is advised, and approaches for early detection of altered pulmonary function, guided by a pulmonary specialist, should be considered. Whether a common relationship exists between sJIA-MAS and interstitial lung disease is unclear. Studies have shown that cytokine storms, allergic reactions caused by biological agents, infections, and other factors in sJIA-MAS are important causes of interstitial lung disease [19]. The present study showed that sJIA-MAS was more likely to involve the interstitial lungs (4/34, 11.8%). Two patients had obvious shortness of breath and chest tightness, and a chest computed tomography revealed consolidation. Pulmonary symptoms and imaging results significantly improved after early corticosteroid treatment, and no deaths occurred. Hypotension occurred in eight patients with MAS, and the incidence of hypotension was as high as 30% in KD-MAS. This finding suggests that hypotension may be an early manifestation of MAS. Guo et al. found that 35% of sJIA patients with MAS had hypotension in a retrospective cohort study, and they also suggested sudden hypotension is an important marker for early identification of MAS [20]. In SLE-MAS, CNS involvement (81.8%) was the most obvious, which was much higher than that reported in the literature. Lerkvaleekul et al. [21] reported that approximately 35% of patients with MAS have CNS dysfunction. As CNS dysfunction in SLE-MAS overlaps with the symptoms of lupus encephalopathy, it is difficult to distinguish between SLE-MAS and lupus encephalopathy. In this study, somnolence, headache, and irritability were classified as CNS involvement, which may be the main reason for the higher proportion of CNS manifestations in the SLE-MAS group than in the sJIA-MAS or KD-MAS groups.

Laboratory changes usually occur before the appearance of the typical clinical symptoms of MAS. In this clinical cohort, compared with the active phase of sJIA, platelet, ESR, and fibrinogen levels were significantly lower in the acute phase of sJIA-MAS, whereas the ferritin, ferritin/ESR, AST, ALT, lactate dehydrogenase, and D-dimer levels were significantly higher. These indicators can predict the occurrence or complete development of MAS in patients with sJIA; however, it is sometimes difficult to distinguish between KD with MAS and refractory KD with drug resistance in clinical practice. Some scholars believe that immunoglobulin-resistant KD is a subclinical hemophagocytosis in nature [22]. Almost all patients with KD-MAS and SLE-MAS had abnormal liver function and elevated ferritin levels. In addition, the white blood cell counts of 54% of patients with SLE-MAS was lower than 3.0 × 10^9^/L. Therefore, we suggest that MAS should be considered when children with lupus have elevated serum ferritin levels, impaired liver function, and a significantly decreased white blood cell count. The ferritin levels and ferritin/ESR ratios of sJIA-MAS were significantly higher than those of KD-MAS and SLE-MAS, suggesting a higher inflammatory state in patients with sJIA-MAS, consistent with the findings of Naveen et al. [23]. We found that the detection rate of bone marrow aspiration phagocytosis in SLE-MAS and KD-MAS was significantly higher than that in sJIA-MAS (81.8% [9/11], 75% [6/8], and 42.4% [14/33], respectively). Routine bone marrow examination suggests that phagocytosis of blood cells by macrophages is strong evidence for the diagnosis of MAS. The positive rate of bone marrow phagocytes in SLE-MAS and KD-MAS was elevated, suggesting that the morphological examination of bone marrow cells is important for the early differentiation of KD-MAS and SLE-MAS.

The random forest model showed that ferritin level, ferritin/ESR, and platelet count had the greatest predictive value for MAS in our study. Eloseily et al. identified that a ferritin: ESR ratio of 21.5 was 82% sensitive and 78% specific for diagnosing sJIA-MAS in large international cohort [24]. In addition, thrombocytopenia has been reported to frequently be the first laboratory discovery of MAS in children [25]. The area under the ROC curve for the combination of these predictors was as high as 0.742, illustrating that the predictors facilitate to early diagnose MAS.

MAS is characterized by a cytokine storm produced by over-activated T lymphocytes and macrophages [25]. The study showed that the serum levels of IL-6 in patients with sJIA-MAS were significantly lower than those in patients with active sJIA, similar to pervious reported literature [26]. More importantly, we found that IL-18 levels were significantly increased at the onset of MAS compared to the active stage of sJIA, suggesting that IL-18 plays an important role in the pathogenesis of MAS. Weiss and Girard-Guyonvarc’h et al. demonstrate the ability of free plasma interleukin-18 (IL-18) to distinguish macrophage activation syndrome (MAS) from other inflammatory disorders [27]. Additionally, with several animal models, they demonstrate that IL-18 is not just a biomarker but rather a driver of inflammation, and is promising as potential therapeutic target in some patients with MAS [28]. A recent study has shown that elevated IL-18 levels can be used as a biomarker of sJIA-MAS [29].

Effective controlled clinical trials of MAS are lacking, and treatment options are mostly based on clinical experience. MAS is usually treated with high-dose corticosteroids (methylprednisolone pulses) combined with a T-cell immunosuppressant (cyclosporin A). In this study, the proportion of sJIA-MAS and SLE-MAS patients treated with methylprednisolone pulse therapy was significantly higher than that in the KD-MAS group, seems that KD complicated with hemophagocytosis is easier to correct clinically. Cyclosporine A plays a key role in the control of inflammatory cytokine storms by blocking NFAT transcription factors and inhibiting cytokine release by T-cells. It was initially administered intravenously and then changed to oral administration after the condition stabilized [1, 30]. In our study, the proportion of cyclosporine used in the three groups of children with MAS was as high as 61–73%. Almost all children with MAS achieved clinical remission after receiving methylprednisolone pulse therapy combined with cyclosporine therapy. In most patients with sJIA-MAS, cyclosporine is used simultaneously with steroid pulse therapy, which is beneficial for the early control of the inflammatory response. IVIG may be effective against KD-MAS or MAS triggered by infection; however, its efficacy is uncertain for most cases of sJIA-MAS [31]. In this study, only 11% of patients with sJIA-MAS were treated with IVIG, whereas 100% of patients with KD-MAS were treated with IVIG, which may be related to the fact that IVIG is the main drug currently used for KD treatment. The choice of immunosuppressive agents in SLE-MAS is controversial, and most rheumatologists prefer cyclosporine to cyclophosphamide because cyclophosphamide can easily cause bone marrow suppression [32]. One patient with SLE-MAS was treated with methylprednisolone pulse therapy combined with Cyclosporine. But in the later stages of the disease, the patient presented with proliferative nephritis, cyclosporine was replaced by cyclophosphamide.

Biological agents for the treatment of immunological diseases and their complications such as MAS have recently become a research hotspot. An IL-1 receptor antagonist (anakinra) can significantly improve the clinical symptoms of sJIA-MAS with failure of traditional treatment, and most patients have clinical symptoms relieved in the early stage of the disease and after increasing the anakinra dose [31, 33]. However, this drug remains in the clinical research stage in China. The IL-6 receptor antagonist, tocilizumab (TCZ), can effectively control systemic inflammation in sJIA. However, some studies have shown that tocilizumab may increase the risk of sJIA-MAS and can mask the increase in C-reactive protein and ferritin when MAS is active, resulting in a delay in MAS diagnosis [19]. Recently, Wu et al. reviewed the cases of 14 children with refractory sJIA-MAS who were treated with TCZ and achieved clinical remission. None of the 14 patients with sJIA-MAS were treated with this monoclonal antibody during an acute attack of hemophagocytosis [34]. Therefore, the treatment of sIJA-MAS with TCZ is still uncertain at present, but it can be used to control systemic inflammation of the primary disease and maintain therapy after MAS is controlled. In this study, four children with sJIA were treated with TCZ for primary disease activity in after MAS was controlled. De Benedetti et al. [35] reported the treatment of 14 patients with sJIA-MAS who had a poor response to cyclosporine or anakinra with the interferon-γ blocking antibody emapalumab, which showed a significant short-term reduction in MAS-related indicators, and all 14 patients survived. Emapalumab may be the most promising monoclonal antibody for treating sJIA-MAS, but it remains unclear whether biological agents are superior to traditional treatments for SLE-MAS and KD-MAS. In this study, a child with SLE-MAS complicated by antiphospholipid syndrome and central retinal artery embolism was treated with rituximab, and the prognosis was good. Junga et al. [36] described a case of MAS secondary to SLE in a young female that responded well to rituximab and highlighted the need for further research on the use of rituximab and other available biologics in the setting of MAS. It should be emphasized that MAS is a broad cytokine storm and blocking individual cytokines may not completely inhibit the cytokine storm triggered during MAS. Biological agents should be used as supplements to traditional treatments for MAS.

There are some limitations about our study. First, owing to the limited number of patients available in our study, the results may be biased. In addition, as a single-center retrospective study, the partial clinical data is subject to missing, and the results need to be validated in a larger sample cohort study. Many multi-sample and multicenter clinical studies are needed in the future.

## Conclusion

MAS is a serious complication secondary to rheumatic diseases and this overlap in clinical presentations makes it challenging to differentiate MAS from active stages of disease. Our study suggests ferritin, ferritin/erythrocyte sedimentation rate and platelet have a high value in predicting sJIA-MAS. In this study, sJIA-MAS is more likely to involve pulmonary interstitial lesions, KD-MAS is more likely to develop KDSS, and SLE-MAS has a higher proportion of central nervous involvement. MAS should be highly suspected in patients with persistent fever who develop hypotension. Elevated ferritin and abnormal liver function have certain significance in suggesting the occurrence of KD-MAS and SLE-MAS. Bone marrow cell morphology examination has high diagnostic value for KD-MAS and SLE-MAS. Our data suggest that IL-18 is markedly elevated in sJIA-MAS and blockade of IL-18 seems to be an effective therapeutic strategy. Methylprednisolone pulse therapy combined with cyclosporine is the most common treatment for MAS. IL-6 antagonist can be used as maintenance treatment for sJIA-MAS when the primary disease is still active after correction.

## Data Availability

The datasets used and/or analyzed in the current study are available and can be obtained from the corresponding author on reasonable request.

## Abbreviations

sJIA: systemic juvenile idiopathic arthritis
KD: Kawasaki disease
SLE: Systemic Lupus Erythematosus
MAS: macrophage activation syndrome
WBC: white blood cell
IVIG: intravenous immunoglobulin
PLT: platelet
Hb: haemoglobin
CRP: C-reactive protein
ESR: erythrocyte sedimentation rate
Alb: albumin
ALT: alanine aminotransferase
AST: aspartate aminotransferase
LDH: lactate dehydrogenase
TG: triglyceride
FIB: fibrinogen
SF: serum ferritin
IQR: interquartile range
CNS: central nervous system
